# Licochalcone B suppresses oxidative stress and apoptosis accompanied by upregulating Nrf2/HO-1 pathway to ameliorate diabetic nephropathy in mice

**DOI:** 10.3389/fphar.2025.1737091

**Published:** 2026-02-10

**Authors:** Shan Luo, Yake Lan, Ruoqi Zhang, Zhongqiu Luan

**Affiliations:** 1 Department of Nephrology, First Affiliated Hospital, Heilongjiang University of Chinese Medicine, Harbin, China; 2 Department of Blood Purification, First Affiliated Hospital, Heilongjiang University of Chinese Medicine, Harbin, China; 3 First Affiliated Hospital, Heilongjiang University of Chinese Medicine, Harbin, China

**Keywords:** apoptosis, diabetic nephropathy, Licochalcone B, mouse, Nrf2/HO-1 pathway, oxidative stress

## Abstract

**Introduction:**

Diabetic nephropathy (DN), as a complication of diabetes, is one of the major causes of end-stage renal disease. Licochalcone B (LCB), a flavonoid active component derived from licorice, is well known for its anti-inflammatory and antioxidant properties. However, the influence of LCB on DN remains unclear. This research investigated the effect of LCB on DN and elucidated the regulatory mechanism.

**Methods:**

We employed male C57BL/6 mice to construct a DN mouse model induced by a high-fat diet (HFD)/streptozotocin (STZ). *In vitro*, a high glucose (HG)-induced injury model in HK-2 (human renal tubular epithelial) cells was used to further confirm the protective effects of LCB.

**Results:**

LCB treatment (20 mg/kg and 40 mg/kg) decreased blood glucose levels, kidney injury, glycogen deposition, and collagen accumulation in the DN mice. Moreover, LCB at a dosage of 40 mg/kg reduced albumin, creatinine, and blood urea nitrogen levels by about 70.7%, 33.4%, and 45.6%, respectively, indicating an improvement in kidney function. In renal tissues, LCB suppressed oxidative stress and apoptosis in HFD/STZ-induced mice. Consistent with *in vivo* findings, LCB alleviated HG-induced oxidative stress and apoptosis in HK-2 cells. Transcriptome analysis revealed that LCB affects oxidative stress and renal function-related pathways to alleviate DN. Further mechanistic studies demonstrated that LCB treatment upregulates the expressions of heme oxygenase-1 (HO-1) and nuclear factor (erythroid-derived 2)-like 2 (Nrf2), suggesting activation of the Nrf2/HO-1 signaling pathway.

**Conclusion:**

Taken together, this research demonstrates that LCB suppresses oxidative stress and apoptosis accompanied by modulating the Nrf2/HO-1 pathway to ameliorate DN, which provides a promising strategy for DN treatment.

## 1 Introduction

Diabetic nephropathy (DN) is a prevalent and severe complication linked to diabetes mellitus and serves as one of the causes of end-stage renal disease (Matoba et al., 2019; Thomas et al., 2016). Globally, the incidence of DN has experienced a significant rise and DN can cause enhanced morbidity and mortality in patients with diabetes mellitus (Samsu, 2021). The early features of DN are glomerular mesangial hyperplasia, basement membrane thickening and glomerulosclerosis, and as the disease progresses, tubulointerstitial fibrosis and tubular atrophy occur, ultimately leading to the progression of end-stage renal disease (Fineberg et al., 2013; Mason and Wahab, 2003). The cardinal pathogenesis of DN encompasses oxidative stress (Brownlee, 2005), the generation and accumulation of advanced glycation end products (Zhou et al., 2012), and chronic inflammation (Navarro-González et al., 2011). Strict control of blood glucose and blood pressure or the administration of angiotensin-converting-enzyme inhibitors or angiotensin-receptor antagonist (Kim et al., 2022; Liu et al., 2022), can mitigate the progression of renal disease, but therapeutic outcomes remain suboptimal. Despite stem cell and gene therapies demonstrating considerable potential to improve outcomes for DN patients, challenges still exist, including tumorigenicity, genomic instability, and restricted direct research (Elendu et al., 2023; Xu et al., 2025a). Hence, the development of novel medicines is of great significance for the treatment of DN.

Licochalcone B (LCB), a bioactive flavonoid compound, is derived from the roots of licorice (*Glycyrrhiza inflata*) (Oh et al., 2019). LCB has been reported that it has pharmacological properties such as anti-inflammatory and antioxidant activity (Fu et al., 2013). LCB exhibited potent anti-inflammatory activity by inhibiting phosphorylation of NF-κB p65 in the LPS signaling pathway (Furusawa et al., 2009). LCB inhibited oxidative stress and inflammation through the Keap1/nuclear factor (erythroid-derived 2)-like 2 (Nrf2) pathways, which in turn ameliorates LPS-induced acute lung injury in mice (Huang et al., 2023a). In addition, LCB is able to bind stably to dipeptidyl peptidase-4 and inhibit DPP-4 at high concentrations, thereby treating type 2 diabetes (Shaikh et al., 2022). Furthermore, LCB can prevent mice from carbon tetrachloride-induced liver injury by suppressing p38 and NF-κB signaling pathways (Kuang et al., 2017; Lin et al., 2017; Teng et al., 2016). Crucially, some scholars investigating the pharmacological activity of wen-pi-tang found that LCB isolated from licorice could inhibit protein glycosylation and speculated that it might be an effective ingredient in the treatment of DN (Nakagawa et al., 2005). However, the role of LCB in DN is still unexplored. Specifically, whether LCB can mitigate glucose-induced damage, proteinuria, and fibrosis in renal cells or animal models, along with its downstream signaling pathways, remain critical questions to be addressed. Therefore, this study aims to systematically evaluate the efficacy of LCB in treating diabetic nephropathy (DN) and to elucidate its molecular mechanisms in depth, thereby filling this specific knowledge gap.

This study aims to systematically evaluate the therapeutic effects of LCB in a mouse model of diabetic nephropathy and to investigate whether it exerts renal protection by activating the Nrf2/HO-1 signaling pathway to suppress oxidative stress and apoptosis.

## 2 Materials and methods

### 2.1 Animal models

A total of thirty-six male C57BL/6 mice (8 weeks old) were randomly divided into four groups: control group (n = 6), HFD/STZ group (n = 6), HFD/STZ+20LCB group (n = 6), and HFD/STZ+40LCB group (n = 6). Mice (5-6 mice per cage) were acclimatized for 1 week under controlled environmental conditions (a 12 h light/dark cycle, temperature of 22 °C ± 1 °C, humidity of 45%–55%). Mice were reared with high-fat diet (HFD; 60% of kcal as fat; D12492 Research Diets, New Brunswick, NJ) for 4 weeks before being intraperitoneally injected with streptozotocin (STZ; dissolved in 0.1 M citrate buffer, pH 4.5) purchased from Macklin at a dosage of 50 mg/kg (Chen et al., 2022) for 5 days. The DN model was constructed and its performance was validated when the fasting blood glucose levels exceeded 16.7 mmol/L. Following STZ administration, mice in the treatment groups received LCB via gavage (20 and 40 mg/kg (Li et al., 2022); dissolved in 5% DMSO, 5% Tween-80, and 90% normal saline) purchased from Shanghai yuanye Bio-Technology Co., Ltd once every 2 days over an eight-week period. Body weight and blood glucose were monitored weekly. Finally, mice were housed in metabolic cages for 24 h to collect urine samples. Afterward, mice were euthanized utilizing isoflurane, and serum and kidney tissues were harvested for subsequent analysis.

### 2.2 Biochemistry assay of the urine and serum samples

The serum concentrations of blood urea nitrogen (BUN), total cholesterol (TC) and triglyceride (TG) were quantified utilizing corresponding kits (Nanjing Jiancheng Bioengineering Institute, Nanjing, China) according to the manufacturer’s instructions. In addition, albumin and creatinine levels in 24-h urine were detected utilizing a mouse ALB ELISA kit (FineTest, Wuhan, China) along with a creatinine assay kit (Nanjing Jiancheng Bioengineering Institute, Nanjing, China) according to the manufacturer’s instructions.

### 2.3 Hematoxylin and eosin (H&E) staining, periodic acid-schiff (PAS) staining and Masson staining

In order to assess histopathologic alterations, renal fibrosis, and glycogen deposition, we carried out H&E, PAS and Masson staining. Briefly, kidney tissues were treated with a series of graded alcohol solutions (70%, 80%, 90% and 100%) to achieve dehydration. The kidney tissues were permeabilized with xylene (Aladdin, Shanghai, China) and embedded in paraffin. After freezing, tissues were sectioned into 5 μm slices. Subsequently, kidney tissue sections were dewaxed and stained with H&E (Solarbio, Beijing, China), Masson staining solution (Leagene, Beijing, China), and glycogen PAS staining solution (Leagene, Beijing, China), respectively. For H&E staining, sections were stained with hematoxylin for 5 min, rinsed, and then stained with eosin for 3 min. For PAS staining, sections were oxidized with a high-iodic acid solution for 10 min in a wet environment, followed by staining with Schiff’s reagent for 15 min. After rinsing for 5 min, hematoxylin is utilized for counterstaining for 2 min. For Masson staining, sections were stained with a Ponceau acid fuchsin staining buffer for 10 min, washed with phosphomolybdic acid solution for 2 min, and then stained with aniline blue solution for 1 min. Finally, a microscope (Olympus, Tokyo, Japan) was utilized for microscopic examination, followed by image acquisition and analysis.

### 2.4 Assessment of apoptosis

TdT-mediated dUTP nick-end labeling (TUNEL) staining was employed to assess apoptosis levels. In brief, paraffin-embedded kidney tissues were made into 5-μm slices and then deparaffinized. Subsequently, the slices were stained utilizing an *in situ* cell detection kit (Roche, Basel, Switzerland) and observed under a microscope (Olympus, Tokyo, Japan). In addition, the activity of caspase-3 and caspase-9 was also determined according to the manufacturer’s instructions by corresponding kits (Solarbio, Beijing, China).

### 2.5 Dihydroethidium (DHE) staining

DHE staining was conducted to determine oxidative stress levels. Cell and tissue slices were incubated with DHE (Beyotime, Shanghai, China) under a dark environment at 37 °C for 30 min. Slices were observed and images were captured under a microscope (Olympus, Tokyo, Japan).

### 2.6 Detection of oxidative stress indicators

In accordance with manufacturer’s protocol, the corresponding kits (Nanjing Jiancheng Bioengineering Institute, Nanjing, China) were employed to determine superoxide dismutase (SOD), catalase (CAT) activities, and malondialdehyde (MDA) content in kidney tissues.

### 2.7 mRNA-sequencing (mRNA-seq)

Extraction of total RNA was conducted from renal tissues in HFD/STZ-induced mice and HFD/STZ-induced mice administered with 40 mg/kg LCB. mRNA was captured from total RNA utilizing poly-T oligo-attached magnetic beads. Then, complementary DNA libraries were generated, and mRNA sequencing (mRNA-seq) was subjected to through the Illumina platform. The criteria for identifying upregulated differentially expressed genes (DEGs) are: fold change (FC) >1.5 and p < 0.05. The criteria for identifying downregulated DEGs are: FC <0.67 and p < 0.05. Enrichment analysis was carried out based on the Gene Ontology (GO) database and Kyoto Encyclopedia of Genes and Genomes (KEGG) database. GO enrichment analysis covering biological processes, molecular functions, and cellular components. The GO and KEGG terms with p < 0.05 were defined as being significantly enriched. The top 15 terms from GO and KEGG are presented.

### 2.8 Cell culture and the establishment of the DN cell model

HK-2 cells provided by iCell were cultured utilizing specialized medium (iCell, Shanghai, China). After 24 h of adherent growth, the medium was substituted with high glucose medium (HG, 35 mmol/L glucose) for 48 h to induce cell injury.

### 2.9 Cell viability

HK-2 cells were plated in 96-well plates at a density of 5 × 10^3^ cells/well. Various concentrations of LCB (1, 3, 6, 12, 25, and 50 μM) were added to treat cells. After 48 h, CCK-8 assay was performed utilizing a CCK-8 cell proliferation detection kit (KeyGEN, Nanjing, China). The OD values at 450 nm were determined. Additionally, cells were cultured with medium containing HG for 48 h and various concentrations of LCB (3, 6, 12, 25, and 50 μM) were utilized to intervene for 48 h, after which cell viability was evaluated via the CCK-8 assay.

### 2.10 Flow cytometry

Cells from each group were harvested through centrifugation at 150 *g* for 5 min after which the supernatant was removed. Cells were resuspended with 500 μL Binding Buffer (KeyGEN, Nanjing, China). Afterwards, cells were stained with 5 μL Annexin V–FITC and 5 μL Propidium iodide (KeyGEN, Jiangsu, China) for 15 min at room temperature in darkness. Eventually, flow cytometry was carried out to measure apoptosis levels.

### 2.11 Real-time PCR

In kidney tissues and HK-2 cells, total RNA was obtained through TRIpure reagent (BioTeke Bio., Beijing, China). cDNA was generated utilizing the all-in-one First-Strand SuperMix kit (Magen, Guangzhou, China). Real-time PCR was carried out on a fluorescent quantitative PCR instrument (Aperbio, Jiangsu, China) to detect the expression levels of heme oxygenase-1 (HO-1). PCR reaction conditions are as follows: pre-denaturation at 95 °C for 5 min, then performing 40 cycles (each cycle containing 10-s denaturation at 95 °C, 10-s annealing at 60 °C, and 15-s extension at 72 °C). The relative change in gene expression levels was quantified employing the 2^−ΔΔCt^ method. The primer sequences designed were as following: mus HO-1 forward: 5′-TGG​TGA​TGG​CTT​CCT​TGT-3′; reverse: 5′-ACC​TCG​TGG​AGA​CGC​TTT-3′; homo HO-1 forward: 5′-TTT​GAG​GAG​TTG​CAG​GAG​C-3′; reverse: 5′-AGG​ACC​CAT​CGG​AGA​AGC-3′.

### 2.12 Immunohistochemical (IHC) staining

Kidney tissue slices were treated with antigen retrieval solution for epitope recovery and incubated with 3% H_2_O_2_ for eliminating endogenous peroxidase activity. After blocking, the slices were incubated with HO-1 antibody (1:100; Proteintech Group, Inc., Rosemont, IL, United States) overnight at 4 °C, and then incubated with HRP-labeled goat anti-rabbit (1:500; Thermo Scientific, Pittsburgh, PA, United States) for 1 h at 37 °C. The slices were stained with DAB and observed under a microscope (Olympus, Tokyo, Japan).

### 2.13 Immunofluorescence (IF)

IF staining was carried out as previously described (Cheng et al., 2016). For kidney tissues, samples were pretreated into 5-μm paraffin-embedded slices prior to staining. For cells, samples were fixed with 4% paraformaldehyde for 15 min and permeabilized with 0.1% Triton X-100 for 30 min prior to staining. Tissue and cell samples were incubated with anti-Nrf2 antibody (1:100, Affinity, Changzhou, China) overnight at 4 °C and then incubated with CY3-conjugated goat anti-rabbit IgG (1:200; Abcam, Cambridge, United Kingdom) for 1 h. Finally, tissue and cell samples were counterstained with DAPI and observed through a microscope (Olympus, Tokyo, Japan).

### 2.14 Western blot

HK-2 cells were lysed with RIPA lysis buffer (Solarbio, Beijing, China) and total protein was harvested by centrifugation at 4 °C (10,000 g) for 5 min. The BCA protein assay kit (Solarbio, Beijing, China) was applied to quantify the protein concentrations. Then, equal amounts of proteins (10–20 μg) were separated through sodium dodecyl sulfate polyacrylamide gel electrophoresis, followed by transfer to polyvinylidene difluoride membranes. After blocking with western blocking buffer (Solarbio, Beijing, China) for 1 h, the membranes were incubated with HO-1 antibody (1:2000, Proteintech Group, Inc., Rosemont, IL, United States) at 4 °C overnight, followed by incubation with HRP-conjugated goat anti-rabbit IgG (1:3,000; Solarbio, Beijing, China) for 1 h at room temperature. Protein bands were visualized with ECL Western blotting substrate (Solarbio, Beijing, China) and optical density was analyzed via the Tanon Image software.

### 2.15 Statistical analysis

All data in this research were presented as mean ± SD. Statistical analysis was carried out through GraphPad Prism 9.0. Differences among multiple groups were assessed through one-way ANOVA with post hoc Tukey’s multiple-comparisons test. Two-way repeated-measures ANOVA with post hoc Tukey’s multiple-comparisons test was performed for analyzing mouse body weight and blood glucose results. Statistical significance was set at a P value <0.05. For *in vivo* animal experiments, “n” represents the number of independent animals. For *in vitro* cell experiments, “n” represents the number of independent experimental replicates.

## 3 Results

### 3.1 LCB is a potential ‘drug-like’ compound

**FIGURE 1:**
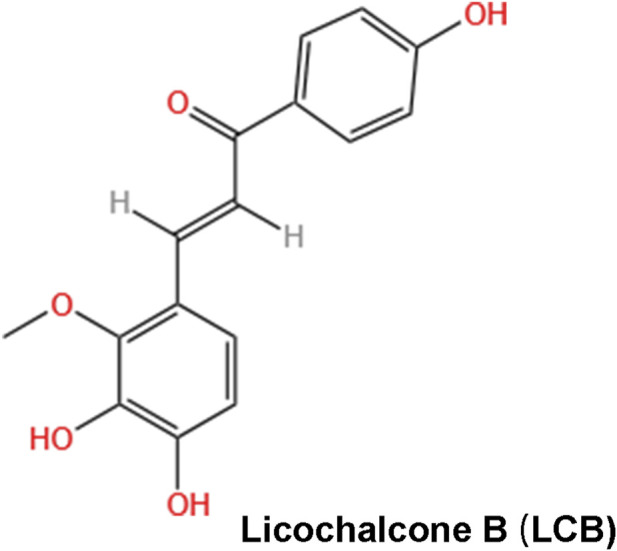
Chemical structure of Licochalcone B (LCB).

We assessed the’drug-like’ the potential of LCB according to Lipinski’s rule. As summarized in Table 1, LCB (Figure 1) exhibits a molecular weight (MW) of 286.28 g/mol and an octanol/water partition coefficient (XLogP3) of 2.6. LCB contains 5 hydrogen-bond acceptors, 3 hydrogen-bond donors and 4 rotatable bonds. These data are obtained from the SwissADME database and comply with Lipinski’s rules. Furthermore, we found that human oral bioavailability (OB) of LCB is 76.76% and drug-likeness (DL) of LCB is 0.19. Given that common drug screening thresholds require OB more than 30% and DL more than 0.18. These results demonstrated that LCB is perceived as’drug-like’ compound.

**TABLE 1:** Pharmacological and molecular characteristics of Licochalcone B (LCB) in SwissADME databases.

Property	Parameter
MW	286.28 g/mol
TPSA	86.99 Å^2^
XLogP3	2.60
Rotatable bonds	4
H-bond acceptors	5
H-bond donors	3
GI absorption	High
BBB permeability	No
Lipinski’s rule	Yes; 0 violations

### 3.2 LCB administration alleviates symptoms of diabetic nephropathy (DN) in HFD/STZ-induced mice

**FIGURE 2:**
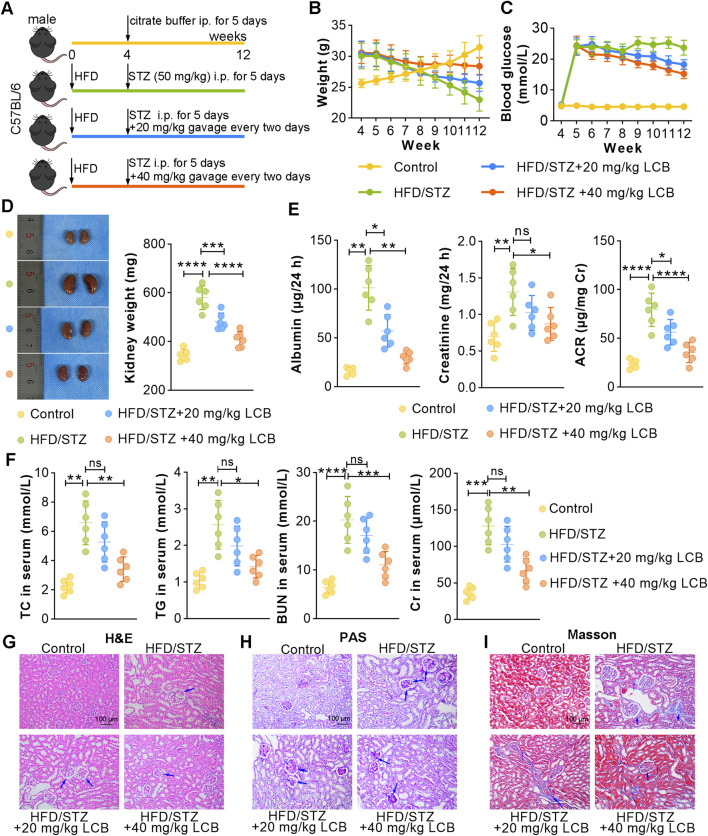
LCB administration alleviates symptoms of diabetic nephropathy (DN) in high-fat diet (HFD)/streptozotocin (STZ)-induced mice. **(A)** Protocol of animal experimental. **(B)** Weight of mice was recorded weekly in each group (n = 6). **(C)** Blood glucose of mice was detected weekly in each group (n = 6). **(D)** Kidney images of each group and kidney weight were recorded (n = 6). **(E)** The albumin and creatinine levels were detected in 24-h urine samples and the ratio of albumin/creatinine (ACR) was calculated (n = 6). **(F)** Serum levels of blood urea nitrogen (BUN), total cholesterol (TC) and triglyceride (TG) and creatinine (Cr) in each group (n = 6). **(G)** Representative histopathological analysis of kidney tissues by Hematoxylin and eosin (H&E) staining in each group (n = 6). Thick arrows indicate glomerular hypertrophy. **(H)** Representative periodic acid-schiff (PAS) staining in kidney tissues of each group (n = 6). Thick arrows indicate basement membrane thickening; thin arrows indicate mesangial expansion. **(I)** Representative Masson staining in kidney tissues of each group (n = 6). Thick arrows indicate interstitial fibrosis; thin arrows indicate renal tubular atrophy. Scale bar, 100 μm. Data are shown as means ± SD. *P < 0.05, **P < 0.01, ***P < 0.001, ****P < 0.0001.

To explore the therapeutic potential of LCB for DN mice (Figure 2A), we first assessed the typical symptoms of DN. As shown in Figures 2B,C, HFD/STZ-induced mice exhibited reduced body weight and elevated blood glucose (fasting blood sugar: 24.12 mmol/L) compared with control mice. After LCB administration, the weights of HFD/STZ-induced mice were enhanced, and the levels of blood glucose were declined. HFD/STZ induction resulted in an increase of the kidney size and weight, LCB treatment reduced the kidney size and weight (Figure 2D). Furthermore, LCB administration could decrease the levels of albumin and creatinine and the ratio of ACR in 24-h urine of HFD/STZ-induced mice (Figure 2E). The results showed that HFD/STZ induction increased the serum levels of TC, TG, BUN and Cr, while LCB administration could downregulate these levels, indicating that LCB can improve lipid metabolic disorder and renal function in DN mice (Figure 2F). Histologically, we further evaluated the impact of LCB on kidney injury by H&E, PAS and Masson staining. We observed increased glomerular area of the kidney in H&E staining in HFD/STZ-induced mice (Figure 2G; Supplementary Figure S2A). The results of PAS staining showed enhanced glycogen expression, thickened basement membrane and mesangial expansion (Figure 2H; Supplementary Figure S2B). Masson staining revealed more collagen deposition, renal tubular atrophy and interstitial fibrosis in HFD/STZ-induced mice (Figure 2I; Supplementary Figure S2C). However, LCB administration reduced kidney injury, glycogen deposition, and accumulation of collagen. Collectively, these findings demonstrated that LCB alleviated kidney injury and improved kidney function of DN mice.

### 3.3 LCB administration mitigates oxidative stress and inhibits apoptosis of kidney tissues in HFD/STZ-induced mice

**FIGURE 3:**
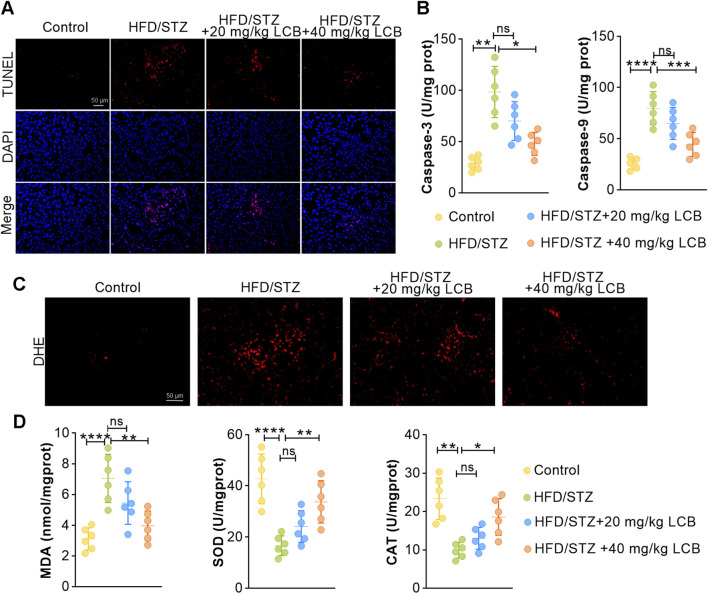
LCB administration mitigates oxidative stress and apoptosis of kidney tissues in HFD/STZ-induced mice. **(A)** Representative TdT-mediated dUTP nick-end labeling (TUNEL) staining in kidney tissues of each group (n = 6). **(B)** The levels of caspase-3 and caspase-9 in kidney tissues of each group (n = 6). **(C)** Representative dihydroethidium (DHE) staining in kidney tissues of each group (n = 6). **(D)** The levels of superoxide dismutase (SOD), catalase (CAT) activities, and malondialdehyde (MDA) in kidney tissues of each group (n = 6). Scale bar, 50 μm. Data are shown as means ± SD. *P < 0.05, **P < 0.01, ***P < 0.001, ****P < 0.0001.

We next investigated the impact of LCB on apoptosis and oxidative stress in renal tissues. TUNEL assay revealed that the apoptotic cells in HFD/STZ-induced mice were increased compared to control mice, while LCB treatment reduced excessive apoptosis (Figure 3A; Supplementary Figure S2D). Meanwhile, we measured the decreased levels of caspase-3 and caspase-9 in HFD/STZ-induced mice treated with LCB, further confirming anti-apoptotic role of LCB (Figure 3B). In addition, we also assessed the impact of LCB on oxidative stress via DHE staining. We observed stronger fluorescence in kidney tissues of HFD/STZ-induced mice, which was reversed by LCB administration (Figure 3C; Supplementary Figure S2E). Consistently, LCB could decrease the levels of MDA and increase the levels of SOD and CAT (Figure 3D). Together, our data suggested that LCB administration mitigated oxidative stress and suppressed apoptosis of kidney tissues in HFD/STZ-induced mice.

### 3.4 Effect of LCB on the renal transcriptome in DN mice

**FIGURE 4:**
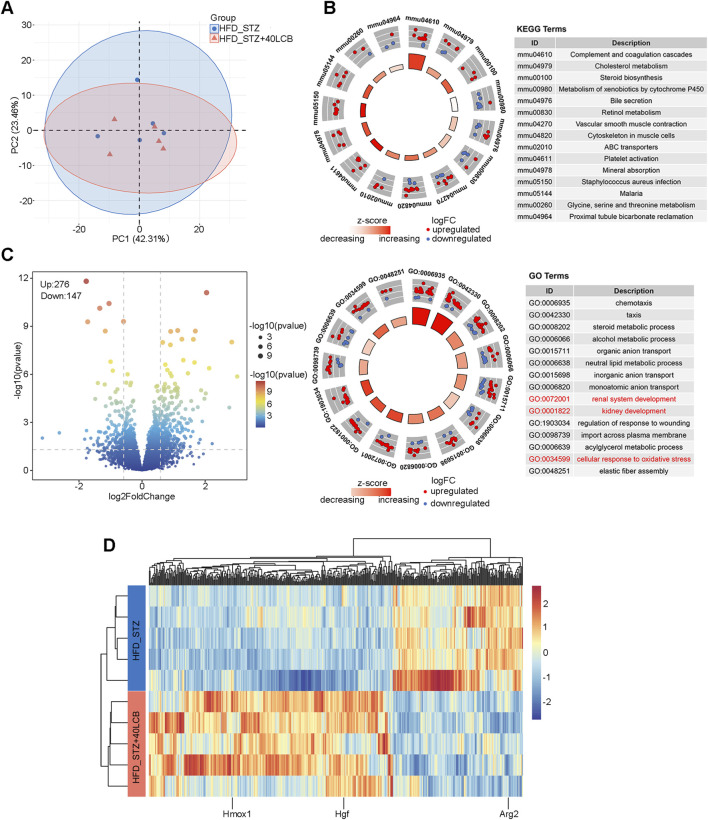
Effect of LCB on the renal transcriptome in DN mice. **(A)** Principal component analysis (PCA) was performed in HFD/STZ and HFD/STZ+40LCB groups. **(B)** Gene Ontology (GO) and Kyoto Encyclopedia of Genes and Genomes (KEGG) enrichment analysis of differentially expressed genes (DEGs) in HFD/STZ and HFD/STZ+40LCB groups. **(C)** Volcano plots of DEGs in HFD/STZ and HFD/STZ+40LCB groups. **(D)** Heatmap of DEGs in HFD/STZ and HFD/STZ+40LCB groups.

In order to investigate the underlying mechanisms, we carried out mRNA-seq to analyze the effects of LCB on the renal transcriptome in DN mice. Principal component analysis (PCA) showed clear distribution of samples in HFD/STZ-induced mice and HFD/STZ-induced mice treated with 40 mg/kg LCB, confirming that internal datasets had high repeatability (Figure 4A). The volcano plot exhibited a total of 423 DEGs including 147 downregulated genes and 276 upregulated genes (Figure 4C). Differences of DEGs levels were displayed in the heatmap (Figure 4D). As shown in Figure 4B, we performed the analysis of GO and KEGG enrichment. The pathways of GO enrichment included renal system development, kidney development and cellular response to oxidative stress, which were related to kidney function and oxidative stress. Protein-protein interaction (PPI) analysis revealed interactions among the factors related to renal system development, kidney development and cellular response to oxidative stress pathways (Supplementary Figure S5). Several genes, including HO-1, Arg2, and Hgf, were associated with these pathways. Herein, we focused on the levels of HO-1were upregulated (FC ≈ 1.51, p < 0.05).

### 3.5 LCB suppresses oxidative stress and apoptosis in HG-induced HK-2 cells

**FIGURE 5:**
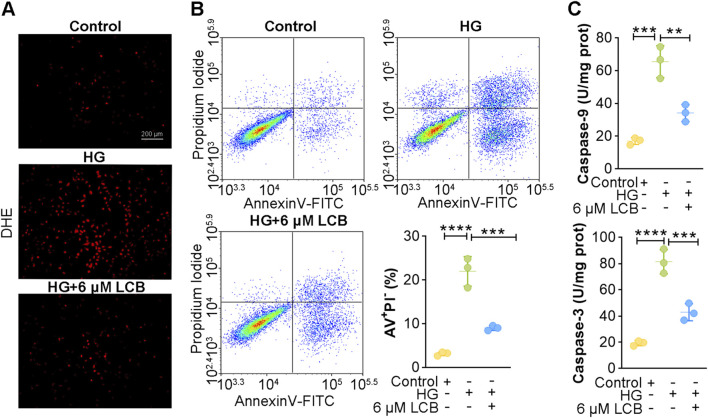
LCB suppresses oxidative stress and apoptosis in high glucose (HG)-induced HK-2 cells. **(A)** Representative DHE staining in HG-induced HK-2 cells of each group (n = 3). **(B)** Apoptosis was detected by flow cytometry (n = 3). **(C)** The levels of caspase-3 and caspase-9 in HG-induced HK-2 cells of each group (n = 3). Scale bar, 200 μm. Data are shown as means ± SD. **P < 0.01, ***P < 0.001, ****P < 0.0001.

We conducted a HG-induced HK-2 cell model to further investigate the cytoprotective effects of LCB on oxidative stress and apoptosis. Firstly, we measured the viability of LCB by CCK8 assay. We found that the LCB concentrations ranging from 1 to 6 μM had no significant effect on HK-2 cell viability (Supplementary Figure S1A). LCB at concentrations of 6, 12, and 25 μM enhanced the viability of HG-induced HK-2 cells (Supplementary Figure S1B). However, LCB concentrations of 12 and 25 μM markedly reduced HK-2 cell viability (Supplementary Figure S1A). Hence, 6 μM LCB was considered the safe and effectively therapeutic concentration for subsequent experiments. The results of DHE staining demonstrated that fluorescence intensity of HG-induced HK-2 cell was stronger than that of control cells, suggesting that HG induction led to the oxidative stress damage. However, LCB treatment markedly alleviated oxidative stress (Figure 5A; Supplementary Figure S3). Results of flow cytometry indicated that HG induction promoted apoptosis, while LCB treatment evidently repressed apoptosis (Figure 5B). This anti-apoptotic effect was further supported by decreased levels of caspase-3 and caspase-9 (Figure 5C). In summary, these findings indicated that LCB alleviated oxidative stress and suppressed apoptosis in HG-induced HK-2 cells.

### 3.6 LCB activates the Nrf2/HO-1 signaling pathway

**FIGURE 6:**
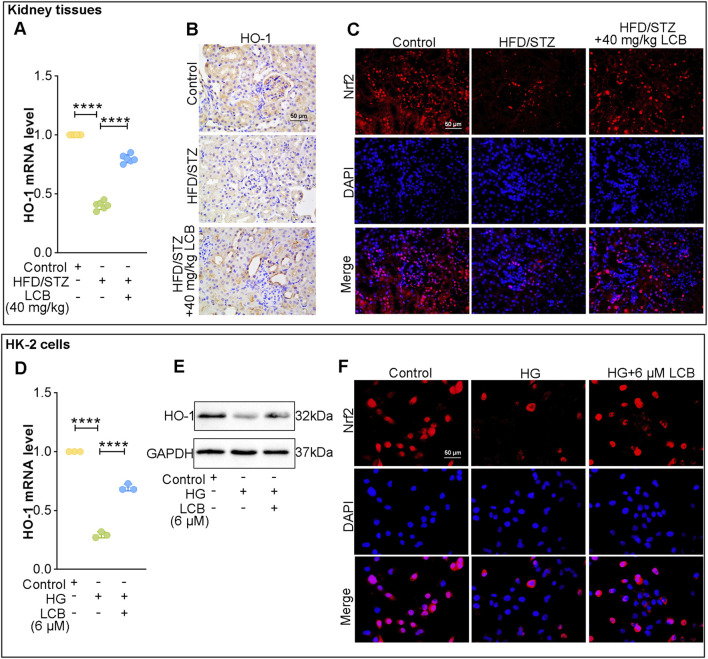
LCB promotes Nrf2/HO-1 pathway. **(A)** The mRNA level of heme oxygenase-1 (HO-1) was assessed through real-time PCR in kidney tissues (n = 6). **(B)** The level of HO-1was assessed through Immunohistochemical (IHC) staining in kidney tissues (n = 6). **(C)** Immunofluorescence (IF) for the assessment of nuclear factor erythroid 2-related factor 2 (Nrf2) in kidney tissues. **(D)** The mRNA level of HO-1was assessed through real-time PCR in HK-2 cells (n = 3). **(E)** The expression level of HO-1was detected through Western blot in HK-2 cells (n = 3). **(F)** IF for the assessment of Nrf2 in HK-2 cells (n = 3). Scale bar, 50 μm. Data are shown as means ± SD. ****P < 0.0001.

We further investigated the impact of LCB on Nrf2/HO-1 pathway. Real-time PCR results indicated that the HO-1 mRNA level significantly increased after LCB intervention compared with HFD/STZ-induced mice (Figure 6A). As depicted in Figure 6B and Supplementary Figures S4A,B, the expression levels of HO-1 were remarkably downregulated in kidney tissues of HFD/STZ-induced mice compared to the control mice, which was reversed by LCB administration. IF staining results revealed that the expressions of Nrf2 were reduced in HFD/STZ-induced mice, while an increase was observed following LCB administration (Figure 6C). Consistently, we found that the expressions of HO-1 and Nrf2 were reduced in HG-induced HK-2 cells, while LCB treatment could enhanced their expression (Figures 6D–F; Supplementary Figures S4C,D; Supplementary Figure S6). Taken together, these results indicated that LCB could activate the Nrf2/HO-1 pathway.

## 4 Discussion

DN is a chronic disease that can lead to end-stage renal disease manifested mainly by albuminuria and declined renal function (Huang et al., 2023b). Effective interventions for treating DN include blood glucose control, blood lipid lowering, and renal function preserving (Selby and Taal, 2020). In recent years, the protective impacts and mechanisms of traditional Chinese medicine and its active ingredients on DN have received extensive attention in the research and development of new drugs (Liu et al., 2022). Glabridin had an ameliorative effect on DN, primarily through modulation of ferroptosis and the VEGF/Akt/ERK pathways (Tan et al., 2022). A previous report has shown that Baicalin is able to reduce oxidative stress and inflammation through the Nrf2 and MAPK pathways, thereby ameliorating DN (Ma et al., 2021). Our study investigated the underlying role of LCB in the progression of DN using HFD/STZ-induced mice. Results indicated that LCB could reduce oxidative stress and suppress apoptosis, thereby improving DN.

The symptoms of DN primarily manifested as the presence of albuminuria (Palmer, 2007). BUN, Cr and ACR are common indicators used to assess renal function (Zhang et al., 2023). The previous research demonstrated that DN mice showed weight loss, decreased blood glucose and upregulated levels of BUN, Cr and ACR (Chen et al., 2022). Notably, LCB mitigated these symptoms of HFD/STZ-induced DN mice. During the development of DN, typical histologic alterations in kidney tissues were glomerulosclerosis, glomerular basement membrane thickening, and expanded mesangium (Selby and Taal, 2020). Our research found that LCB could improve these histologic alterations. Improvement of renal function is considered to be associated with the reduction of blood lipids (Zheng et al., 2021). Diabetes can cause dyslipidemia, and high blood lipids may lead to glomerulosclerosis in DN (Selby and Taal, 2020). TG and TC are lipid biomarkers used to assess the levels of lipid metabolism (Deng et al., 2022). Elevated TG levels are thought to be a contributing factor in the development of proteinuria (Hwang et al., 2024). In our study, we found that DN mice showed high levels of TG and TC, while LCB treatment could mitigate the levels of blood lipid.

Oxidative stress is considered to be the key cause of the onset and development of DN (Tavafi, 2013). Hyperglycemia results in massive ROS production, further increasing oxidative stress (Jin et al., 2023). Increased oxidative stress causes metabolic alterations of kidney tissues and alterations of kidney hemodynamics, ultimately leading to physiological and pathological damage of kidney tissues (Hernandez et al., 2022). Excessive ROS production elevates TGF-β1 and NF-κB levels, leading to tubulointerstitial fibrosis in DN patients (Dwivedi and Sikarwar, 2025). It is thus speculated that the inhibition of oxidative stress by LCB leads to the suppression of the TGF-β1 and NF-κB pathways, ultimately reducing tubulointerstitial fibrosis via the inhibition of fibroblast activation. Furthermore, oxidative stress can activate apoptosis pathways, which in turn lead to kidney injury (Jaikumkao et al., 2024). Caspase-3 and caspase-9 are critical mediators of apoptosis, and their activation promotes apoptosis (Unnisa et al., 2023). In the current study, we found high levels of caspase-3 and caspase-9 in kidney tissues of DN mice, while LCB treatment significantly downregulated the levels of caspase-3 and caspase-9. Consistently, the results of *in vitro* experiments also demonstrated that LCB was able to reduce caspase-3 and caspase-9 levels in HG-induced HK-2 cells. These results indicated that LCB inhibits oxidative stress, thereby suppressing the activation of caspase-3 and caspase-9, ultimately inhibiting apoptosis.

To further explore the underlying regulatory mechanisms of LCB to ameliorate DN, we performed transcriptome analysis in renal tissues of DN mice. Enrichment results indicated that DEGs involved in renal function and oxidative stress pathways included HO-1, Arg2, Hgf, Nr4a2, Nid1, Fbn1, Fabp1, Robo1 and so on. It has been reported that silencing Arg2 is able to improve DN (Morris et al., 2011). Hgf gene therapy is also effective in improving DN (Cruzado et al., 2004). Nrf2 is considered to be a regulator of oxidative stress responses, and activation of Nrf2 signaling can modulate the expression of HO-1 (antioxidant genes) and inhibit oxidative damage thereby improving DN (Hasan et al., 2025; Hashemi et al., 2023). Herein, we hypothesized that LCB exerted the improvement roles by regulating the Nrf2/HO-1 pathway. Consistently, results of *in vitro* experiments showed that LCB treatment upregulated Nrf2 levels and increased HO-1 expression. These findings suggest that activating the Nrf2/HO-1 pathway and suppressing oxidative stress through this mechanism may represent a potential strategy for preventing tubulointerstitial fibrosis in DN. Additionally, Nr4a2, Nid1, Fbn1, Fabp1, and Robo1 have also been reported to participate in regulating DN progression (Abo El-Asrar et al., 2023; Khattab and Torkamani, 2022; Liang et al., 2022; Liu et al., 2018; Xu et al., 2025b). This suggests that Arg2, Hgf, Nr4a2, Nid1, Fbn1, Fabp1, and Robo1 may also serve as potential targets for LCB, warranting further validation in future studies.

This research has several limitations. Firstly, the relatively short duration of the treatment may not fully capture the long-term therapeutic effects or potential adverse outcomes. Secondly, the research lacks the confirmation of protein levels through Western blot experiments. The findings derived from animal models require further validation to assess their direct translation to human pathophysiology. In addition, our work explores the protective role of LCB in DN using only a single cell line. It is essential to investigate in other cell lines. Finally, whether LCB suppresses oxidative stress and apoptosis by modulating the Nrf2/HO-1 pathway requires further validation to strengthen the mechanistic rigor.

## 5 Conclusion

In summary, this study demonstrated that LCB had the underlying protective role against DN in HFD/STZ-induced mice. LCB could reduce kidney injury, glycogen deposition, and accumulation of collagen in DN mice. LCB could reduce the levels of albumin, creatinine, BUN, TG and TC, suggesting that LCB improves the kidney function of DN mice. Furthermore, LCB could suppress oxidative stress and apoptosis in kidney tissues of HFD/STZ-induced mice and HG-induced HK-2 cells, thereby mitigating DN. The results of mRNA-seq indicated that oxidative stress-related pathways are involved in the improvement of DN by LCB, and the expression of the related factor HO-1 is significantly upregulated. Further studies verified that LCB treatment upregulates the expressions of HO-1 and Nrf2, indicating that LCB mitigates DN accompanied by modulating the HO-1/Nrf2 pathway. This is one of the possible regulatory mechanisms by which LCB improves DN.

## Data Availability

The data presented in the study are available at the Dryad repository: 10.5061/dryad.zkh1893r3.
