# Supplementary material for: Licochalcone B suppresses oxidative stress and apoptosis accompanied by upregulating Nrf2/HO-1 pathway to ameliorate diabetic nephropathy in mice
Source: Front Pharmacol. 2026 Feb 10;16:1737091. doi: 10.3389/fphar.2025.1737091 (PMC12929500; doi:10.3389/fphar.2025.1737091)
Supplement: Supplementary file 1 [file Supplementaryfile1.docx]

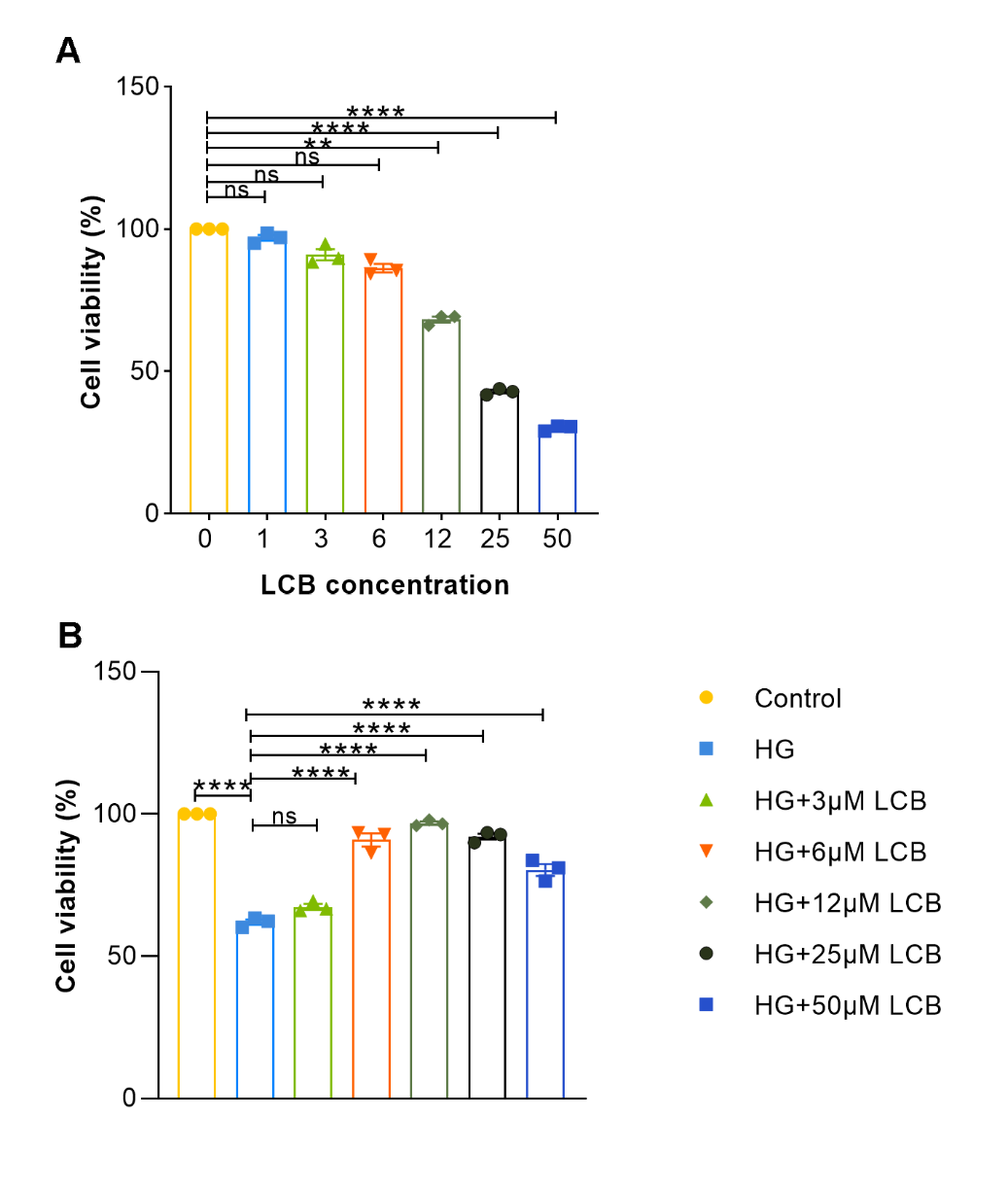


**Supplementary Figure 1. Screening for LCB therapeutic concentrations.**

(A) HK-2 cells were treated with different concentrations of LCB (0, 1, 3, 6, 12, 25, and 50 µM) for 24 h, and cell viability was detected by CCK-8 (n=3). (B) The cells were treated with HG and different concentrations of LCB (0, 3, 6, 12, 25, and 50 µM) for 48 h. Cell viability was then measured by CCK-8 (n=3). Data are shown as means ± SD. ** P < 0.01, **** P < 0.0001.


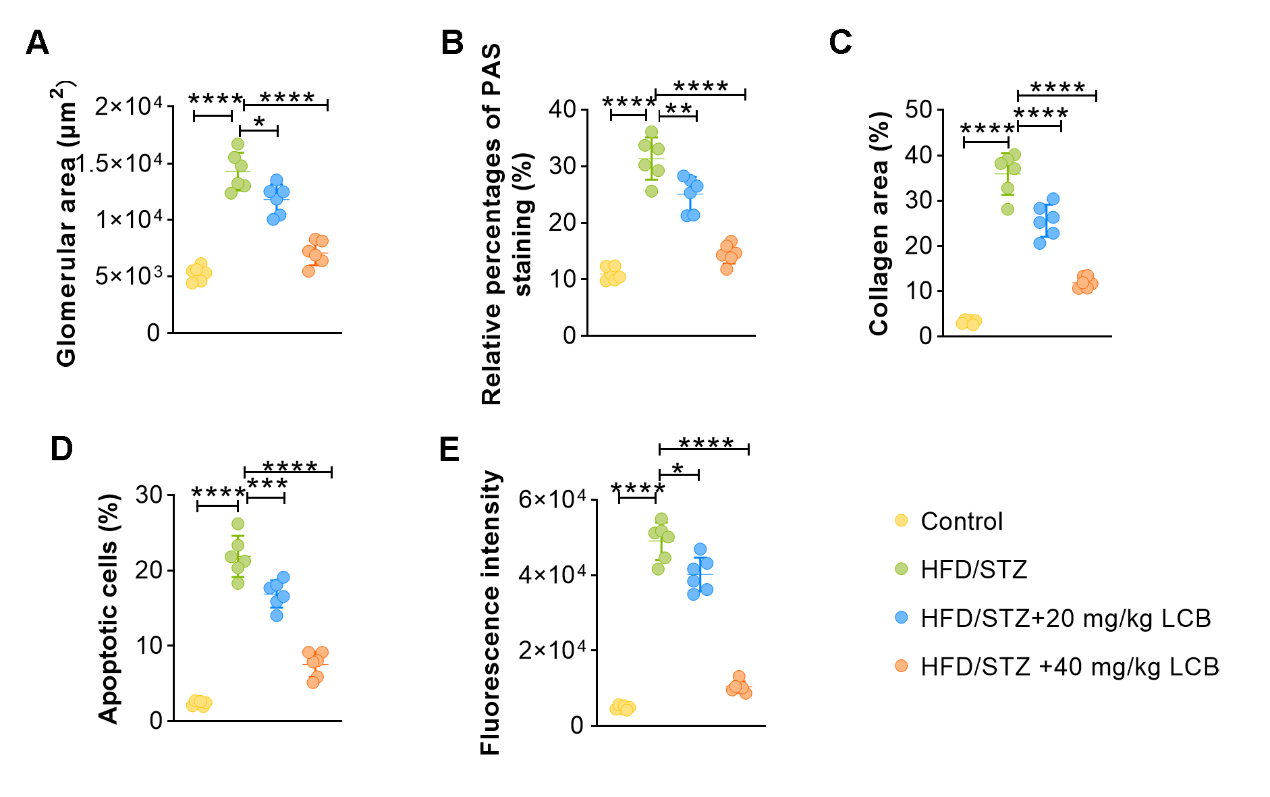


**Supplementary Figure 2. Quantitative Analysis of Histological Examination.**

(A) Glomerular area was quantified in H&E staining (n=6). (B) Glycogen deposition was quantified in PAS staining (n=6). (C) Collagen area was quantified in Masson staining (n=6). (D) The percentage of apoptotic cells was counted (n=6). (E) Fluorescence intensity was quantified in DHE staining (n=6).


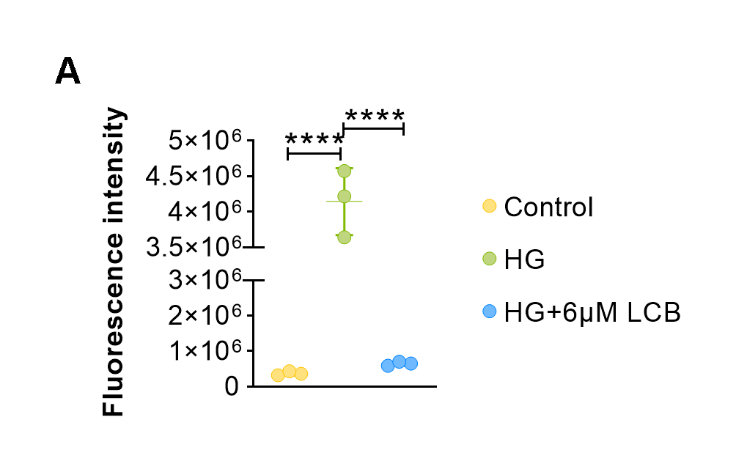


**Supplementary Figure 3. Quantitative Analysis of DHE staining in HG-induced HK-2 cells.**

(A) Fluorescence intensity was quantified in DHE staining (n=3).


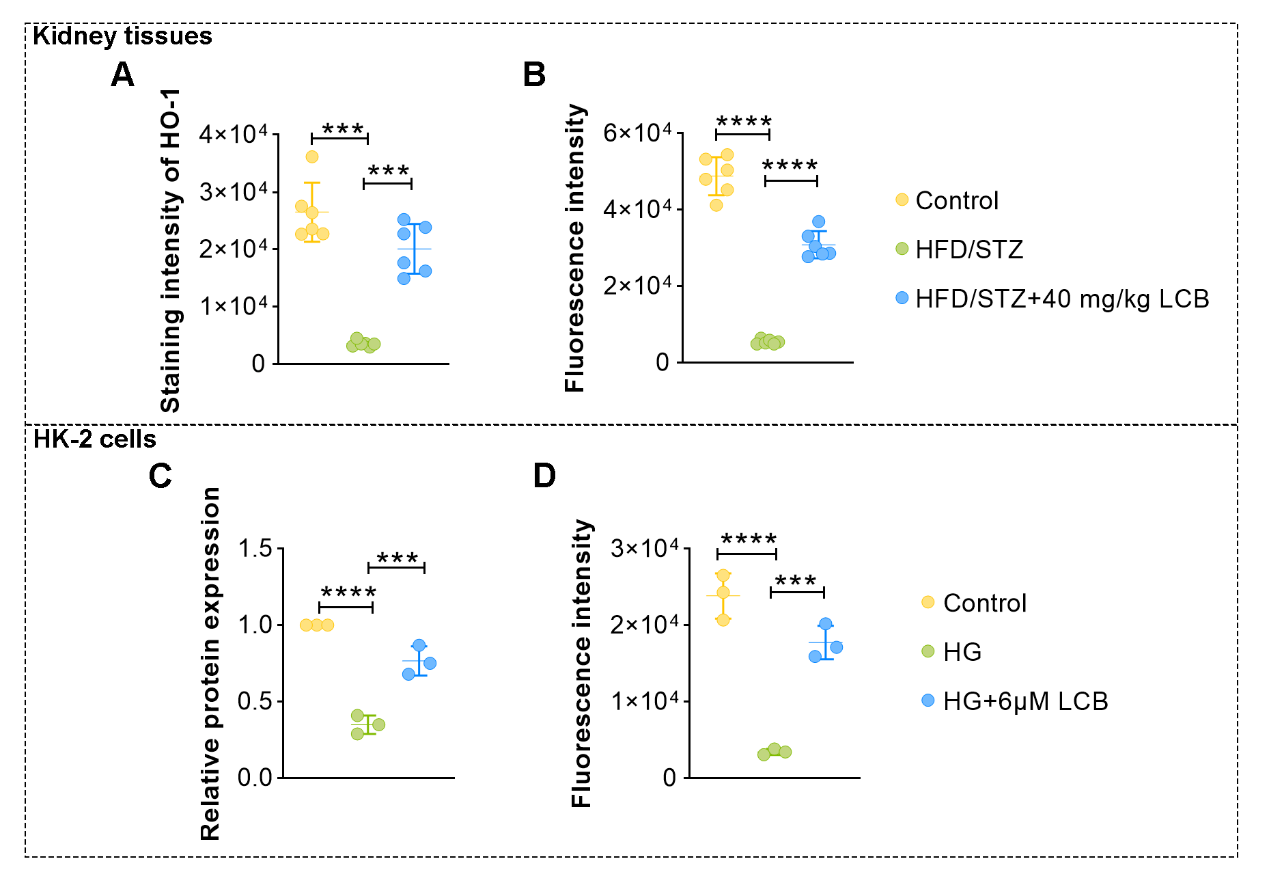


**Supplementary Figure 4. Quantitative Analysis of IHC and IF staining in Figure 6.**

(A) HO-1 staining intensity was quantified in IHC staining. (n=6). (B) Fluorescence intensity was quantified in IF staining (n=6). (C) HO-1 protein levels were quantified. (n=3). (D) Fluorescence intensity was quantified in IF staining (n=3).


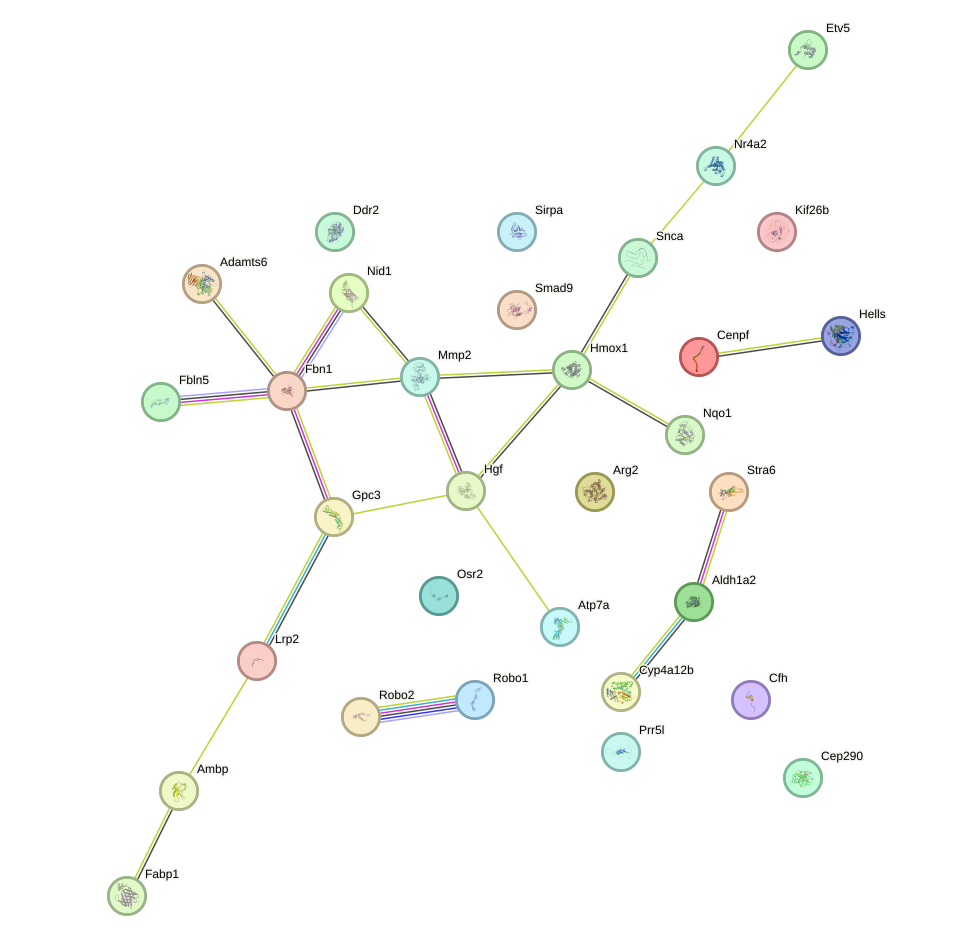


**Supplementary Figure 5. The PPI network analysis of factors related to renal system development, kidney development and cellular response to oxidative stress pathways.**


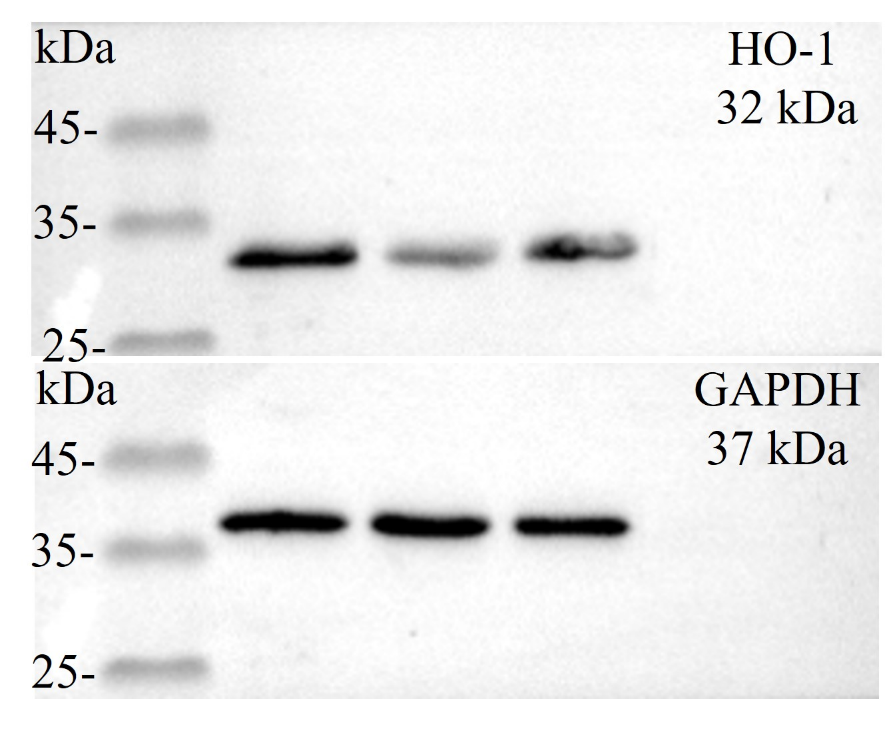


**Supplementary Figure 6. The original image of Figure 6E.**
